# Large-scale validation of methods for cytotoxic T-lymphocyte epitope prediction

**DOI:** 10.1186/1471-2105-8-424

**Published:** 2007-10-31

**Authors:** Mette V Larsen, Claus Lundegaard, Kasper Lamberth, Soren Buus, Ole Lund, Morten Nielsen

**Affiliations:** 1 Center for Biological Sequence Analysis, BioCentrum-DTU, Building 208, Technical University of Denmark, DK-2800 Lyngby, Denmark; 2 Institute for Medical Microbiology and Immunology, Panum Institute 18.3.12, Blegdamsvej 3, DK-2200 Copenhagen N, Denmark

## Abstract

**Background:**

Reliable predictions of Cytotoxic T lymphocyte (CTL) epitopes are essential for rational vaccine design. Most importantly, they can minimize the experimental effort needed to identify epitopes. NetCTL is a web-based tool designed for predicting human CTL epitopes in any given protein. It does so by integrating predictions of proteasomal cleavage, TAP transport efficiency, and MHC class I affinity. At least four other methods have been developed recently that likewise attempt to predict CTL epitopes: EpiJen, MAPPP, MHC-pathway, and WAPP. In order to compare the performance of prediction methods, objective benchmarks and standardized performance measures are needed. Here, we develop such large-scale benchmark and corresponding performance measures and report the performance of an updated version 1.2 of NetCTL in comparison with the four other methods.

**Results:**

We define a number of performance measures that can handle the different types of output data from the five methods. We use two evaluation datasets consisting of known HIV CTL epitopes and their source proteins. The source proteins are split into all possible 9 mers and except for annotated epitopes; all other 9 mers are considered non-epitopes. In the RANK measure, we compare two methods at a time and count how often each of the methods rank the epitope highest. In another measure, we find the specificity of the methods at three predefined sensitivity values. Lastly, for each method, we calculate the percentage of known epitopes that rank within the 5% peptides with the highest predicted score.

**Conclusion:**

NetCTL-1.2 is demonstrated to have a higher predictive performance than EpiJen, MAPPP, MHC-pathway, and WAPP on all performance measures. The higher performance of NetCTL-1.2 as compared to EpiJen and MHC-pathway is, however, not statistically significant on all measures. In the large-scale benchmark calculation consisting of 216 known HIV epitopes covering all 12 recognized HLA supertypes, the NetCTL-1.2 method was shown to have a sensitivity among the 5% top-scoring peptides above 0.72. On this dataset, the best of the other methods achieved a sensitivity of 0.64. The NetCTL-1.2 method is available at .

All used datasets are available at .

## Background

The CTLs of the immune system must be able to discriminate between healthy and infected cells, since only the infected cells are to be eliminated. To facilitate the discrimination, all nucleated cells present a selection of the peptides contained in their proteins on the cell surface in complex with Major Histocompatibility Complex class I (MHC class I) molecules. The course of events leading to MHC class I presentation includes the ongoing degradation of the cell's proteins by the proteasome [1-5]. A subset of the generated peptides are then transported into the Endoplasmatic Reticulum (ER) by Transporter associated with Antigen Presentation (TAP) molecules [6-8]. Once inside the ER, the peptides may bind to MHC class I molecules, which are subsequently transported to the cell surface, where the complexes may be recognized by passing CTLs. The most restrictive step involved in antigen presentation is binding of the peptide to MHC class I. It is estimated that only 1 out of 200 peptides will bind a given MHC class I allele with sufficient strength to elicit a CTL response [9]. However, also proteasomal cleavage and TAP transport efficiency show some degree of specificity [4,9].

Reliable predictions of immunogenic peptides can minimize the experimental effort needed to identify new epitopes to be used in, for example, vaccine design or for diagnostic purposes. We have previously described a method, NetCTL (hereafter renamed NetCTL-1.0), which integrates the predictions of proteasomal cleavage, TAP transport efficiency, and MHC class I affinity to an overall prediction of CTL epitopes [10]. In the following, we describe an improved version of NetCTL, version 1.2. Several other groups have likewise attempted to generate methods that enable CTL epitope identification. On an independent evaluation dataset of known HIV CTL epitopes, NetCTL-1.0 has previously been shown to have a higher predictive performance than the publicly available SYFPEITHI Epitope Prediction method [11,12] and the BIMAS HLA Peptide Binding Prediction method [13,14]. Here, we compare the performance of NetCTL-1.2 to four other publicly available methods, which have been described within the last few years: MAPPP [15], which combines proteasomal cleavage predictions with MHC class I affinity predictions, and EpiJen [16], MHC-pathway [17,18], and WAPP [19], which operate with predictions of both proteasomal cleavage, TAP transport efficiency, and MHC class I affinity. Even for skilled scientist within the field it is not straightforward to compare the performance of the various methods, since they do not necessarily have the same output format and do not cover the same output range. In addition, many different performance measures can be applied, but not all are equally well suited for every method. It is also important to keep in mind that some performance measures are not meaningful on their own. An example of the latter is the performance measure *sensitivity*. In the case of finding CTL epitopes among a large number of peptides, sensitivity is defined as the number of peptides correctly predicted to be CTL epitopes (also called the number of True Positives, TP) divided by the total number of CTL epitopes in the dataset (also called Actual Positives, AP). A method, which finds all CTL epitopes, has a sensitivity of 1. This performance can, however, easily be achieved if the method predicts every peptide to be a CTL epitope. Obviously, such a method is totally useless. In this study, we have defined a number of performance measures, which together give an objective assessment of the methods. On all measures, we find that NetCTL-1.2 has a higher predictive performance than EpiJen, MAPPP, MHC-pathway, and WAPP, although when comparing NetCTL-1.2 with EpiJen and MHC-pathway, the higher predictive performance of NetCTL-1.2 is not statistically significant on all measures.

## Results

### NetCTL-1.2

NetCTL predicts CTL epitopes by integrating predictions of proteasomal cleavage, TAP transport efficiency, and MHC class I binding [10]. Version 1.2 is an improvement on several accounts. Firstly, it predicts epitopes restricted to the A26 and B39 supertypes thus completing the list of 12 recognized supertypes [20]. Secondly, it has an improved performance as compared to the older version 1.0. This is partly due to the use of newer methods for predicting MHC class I affinity and proteasomal cleavage. Furthermore, a larger dataset has been used to deduce the optimal weights on proteasomal cleavage, TAP transport efficiency, and MHC class I affinity, respectively. When testing the performance of NetCTL-1.0 versus NetCTL-1.2 on the independent HIV evaluation dataset consisting of 216 known CTL epitopes, NetCTL-1.0 has an average AUC (Area Under the ROC Curve) per epitope-protein pair of 0.931, while NetCTL-1.2 has an average AUC per epitope-protein pair of 0.941. This difference in predictive performance between NetCTL-1.0 and NetCTL-1.2 is significant at P = 0.02 (paired t-test). For comparison, NetMHC-3.0^NO_HIV^, which is the MHC class I affinity predictor used in NetCTL-1.2, has an average AUC per epitope-protein pair of 0.922. The difference in predictive performance between NetCTL-1.2 and NetMHC-3.0^NO_HIV ^is significant at P = 0.004 (paired t-test).

### Comparing different methods for CTL epitope prediction by using the AUC value

We wanted to compare the performance of NetCTL-1.2 to that of four other publicly available CTL epitope prediction methods: EpiJen [16], MAPPP [15], MHC-pathway [17,18], and WAPP [19]. For the comparisons, we use two evaluation sets containing experimentally verified HIV CTL epitopes and their source proteins: The HIV dataset, which we compiled ourselves, contains 216 epitope-protein pairs restricted to all 12 recognized supertypes. When comparing the performance of NetCTL-1.2 to that of any of the other four methods, only the subset of supertypes also covered by the test method is included. The other dataset is called HIV^EpiJen^. It was taken almost in complete from [16] and contains 87 epitopes restricted to the A1, A2, or A3 supertypes. All five methods can perform predictions for these three supertypes.

**Figure 1:**
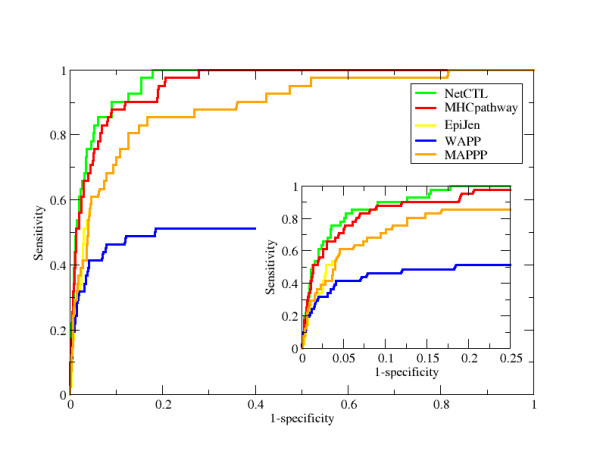
**ROC curves**. The analysis has been performed on 41 A3 restricted epitope-protein pairs from the HIV dataset.

In the above section, we used the AUC value to compare NetCTL-1.2 to NetCTL-1.0 and NetMHC-3.0^NO_HIV^. This measure is, however, not appropriate for the EpiJen and WAPP methods. These methods do not produce a single, combined score for each peptide in the dataset. Instead, the proteasomal cleavage and TAP transport predictors act as filters that reduce the number of possible epitopes. In addition, the EpiJen server maximally outputs the 5% peptides, which have the highest predicted MHC class I affinity and at the same time pass the proteasomal cleavage and TAP transport filters. The problem is exemplified in the ROC (Receiver Operating Characteristic) curve shown in Figure 1. For NetCTL-1.2, MAPPP, and MHC-pathway, the combined score is used as the predicted value. For EpiJen and WAPP, we used the predicted MHC class I affinity as the predicted value. The ROC curves for the two last-mentioned methods come to an abrupt stop, since there are no predicted values for peptides that do not pass the proteasomal cleavage and TAP transport filters. The ROC curves also highlight the need for extracting sensitivity at comparable specificity levels and vice versa in order to achieve objective benchmark comparisons between different methods: Any of the methods can be assigned the highest sensitivity, if the specificity is not set at a comparable level.

### The RANK measure

**Figure 2:**
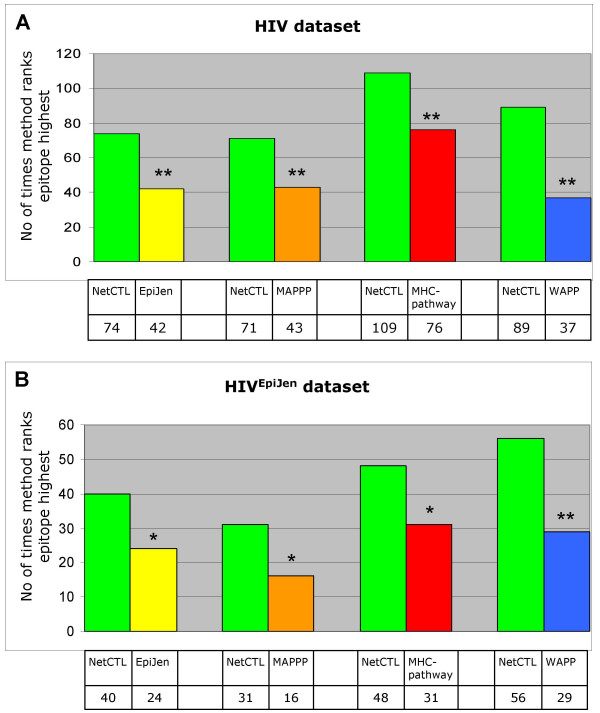
**Performance on the RANK measure**. For each epitope-protein pair, the rank that is assigned to the epitope when using NetCTL-1.2 is compared to the rank assigned when using the test method (EpiJen, MAPPP, MHC-pathway, or WAPP). The height of the bars indicates how often, respectively, NetCTL or the test method ranks the epitope highest. **A: **The HIV dataset has been used for the analysis. When comparing NetCTL-1.2 to either of the test methods, only predictions for supertypes that the test method covers are included. **B: **The HIV^EpiJen ^dataset has been used for the analysis. ** The difference is significant at P < 0.01. * The difference is significant at P < 0.05.

Since the AUC measure is not applicable to all methods, we designed a new measure, which we call the RANK measure. Looking at each epitope-protein pair separately for either the HIV or HIV^EpiJen ^dataset, we rank all possible 9 mers according to the prediction score of a given method. Next, we compare two methods at a time: NetCTL-1.2 and one of the four test methods (EpiJen, MAPPP, MHC-pathway, or WAPP). Again, we use the combined score as the predicted value for NetCTL-1.2, MAPPP, and MHC-pathway, and the predicted MHC class I affinity for EpiJen and WAPP. We then count how often NetCTL-1.2 ranks the epitope higher than the test method, and vice versa. To facilitate a fair comparison to the EpiJen and WAPP methods, where predictions are limited to a subset of the peptides, only the top N of the NetCTL-1.2 predictions are included, where N is the number of peptides assigned a prediction score by the test method (EpiJen or WAPP). All peptides without a predicted value are assigned the rank 9999 to put them at the bottom of the rank-list. In this way, all methods are compared on an equal number of peptide data. Figure 2 shows the results. In Figure 2A, it is seen that for all comparisons, NetCTL-1.2 more frequently ranks the epitope higher than any of the four test methods on the HIV dataset. The difference is significant at P < 0.01 (Binomial test). In Figure 2B, the results are shown for the HIV^EpiJen ^dataset. Also here, NetCTL-1.2 more frequently ranks the epitope higher than the test method. For WAPP the difference is significant at P < 0.01, while for EpiJen, MAPPP, and MHC-pathway the difference is significant at P < 0.05 (Binomial test).

### Specificity at a predefined sensitivity

**Figure 3:**
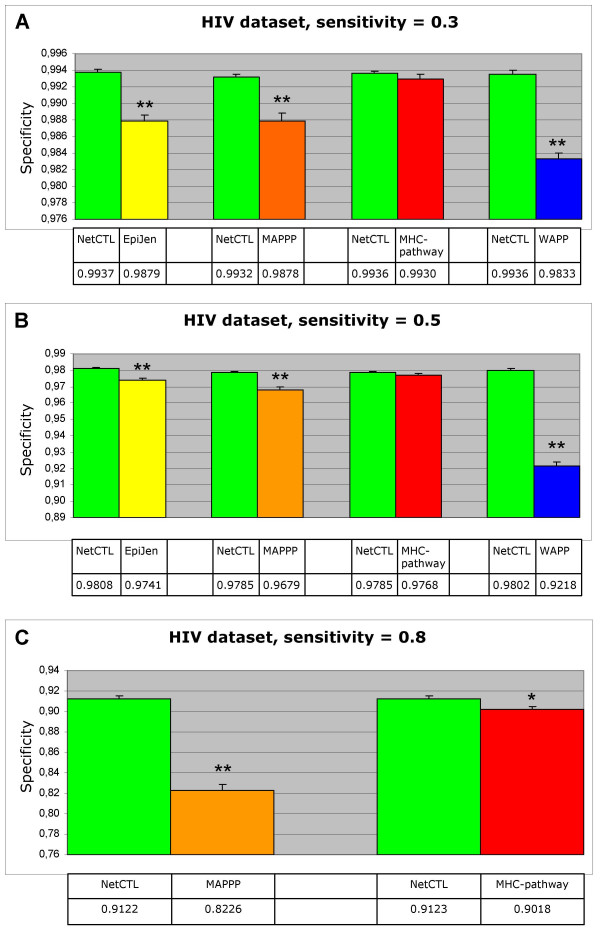
**Comparing specificities**. The HIV dataset has been used for the analysis. In order to include epitopes restricted to as many supertypes as possible, NetCTL-1.2 is compared to each of the other methods separately. For each comparison, only predictions for supertypes that the test method covers are included. The average specificity is found at a predefined average sensitivity using either NetCTL-1.2 or one of the four test methods (EpiJen, MAPPP, MHC-pathway, WAPP). **A: **Average sensitivity = 0.3, **B: **Average sensitivity = 0.5, **C: **Average sensitivity = 0.8. Only NetCTL-1.2, MAPPP and MHC-pathway provide enough predicted scores to obtain a sensitivity of 0.8. The error bars are the standard error. ** The difference is significant at P < 0.01. * The difference is significant at P < 0.05.

When using the default settings at the NetCTL-1.2, MAPPP, and WAPP servers, thresholds are defined that separate the predicted epitopes from the predicted non-epitopes. At the EpiJen server, one can choose between defining the top-scoring 5%, 4%, 3%, or 2% peptides as epitopes. MHC-pathway does as yet not offer any thresholds for separating predicted epitopes from non-epitopes. These differences pose a challenge when comparing the performance of the methods as regards to sensitivity and specificity, since it is a prerequisite for the calculation of these measures that the predicted epitopes can be separated from the non-epitopes. Furthermore, as mentioned earlier, it is generally problematic to distinguish which method has the highest predictive performance, if one method has the highest sensitivity, while the other method has the highest specificity. To overcome these problems, we chose to compare the specificity of the methods at a series of predefined sensitivity values. We chose three predefined sensitivities: 0.3, 0.5, and 0.8. For the HIV dataset, we again compared two methods at a time: NetCTL-1.2 and one of the four test methods, in order to include epitopes restricted to as many supertypes as possible. For the HIV^EpiJen ^dataset, all methods can be compared simultaneously, since all methods can predict epitopes restricted to the A1, A2, and A3 supertypes. We first identified the prediction threshold values that result in the desired sensitivity when averaging over all epitope-protein pairs. We then used the same thresholds to find the average specificity. Figure 3 shows the results for the HIV dataset. It can be seen that NetCTL-1.2 has a significantly higher specificity than EpiJen, MAPPP, and WAPP at all sensitivities (P < 0.01, unpaired student's t-test). When comparing NetCTL-1.2 to MHC-pathway, it can be seen that at an average sensitivity of 0.3 and 0.5 NetCTL has a higher specificity than MHC-pathway although this difference is not statistically significant. At an average sensitivity of 0.8, NetCTL-1.2 has significantly higher specificity than MHC-pathway (P < 0.05, unpaired student's t-test).

When using the HIV^EpiJen ^dataset for the analysis, NetCTL-1.2 has a higher specificity than all the test methods at all sensitivities, although for EpiJen and MHC-pathway the difference is not statistically significant at all sensitivities (the results are available as supplementary material at [21]).

### Sensitivity among the 5% top-scoring peptides

For an experimentalist who wants to find epitopes in a specific protein, it is interesting to know how many of the actual epitopes one can expect to find if testing a certain top-fraction of the peptides. For this, we calculate the sensitivity among the 5% top-scoring peptides. For the HIV dataset, we made the calculations for NetCTL-1.2 and one of the four test methods at a time. For the HIV^EpiJen ^dataset, all methods could be compared using the same dataset, since all methods can predict epitopes restricted to the A1, A2, and A3 supertypes. Table 1 and 2 show the results. Table 1 shows that when NetCTL-1.2 is compared separately to either of the test methods using the HIV dataset, NetCTL-1.2 has the highest sensitivity among the 5% top-scoring peptides with sensitivity values in the range of 0.70–0.78 depending on the evaluation dataset. When evaluating on the HIV^EpiJen ^dataset (Table 2) NetCTL-1.2 also achieves the highest sensitivity of 0.75. On this dataset, MAPPP achieves the second highest sensitivity (0.64), closely followed by MHC-pathway (0.63). EpiJen achieves a sensitivity of 0.60, while WAPP only achieves a sensitivity of 0.44 among the 5% top-scoring peptides.

**Table 1:** Determining the sensitivity among the 5% top-scoring peptides on the HIV dataset

	NetCTL-1.2	EpiJen	NetCTL-1.2	MAPPP	NetCTL-1.2	MHC-pathway	NetCTL-1.2	WAPP
			
HIV	0.72	0.63	0.70	0.57	0.70	0.64	0.78	0.44

The HIV dataset has been used for the analysis. To be able to include epitopes restricted to as many supertypes as possible, NetCTL-1.2 is compared to each of the other methods separately. For each comparison, only predictions for supertypes covered by the test method are included.

**Table 2:** Determining the sensitivity among the 5% top-scoring peptides on the HIV^EpiJen ^dataset

	NetCTL-1.2	EpiJen	MAPPP	MHC-pathway	WAPP
HIV^EpiJen^	0.75	0.60	0.64	0.63	0.44

The HIV^EpiJen ^dataset has been used for the analysis. All methods can be compared simultaneously since this dataset only contains epitopes restricted to the A1, A2, or A3 supertypes, which all methods cover.

## Discussion

Reliable CTL epitope predictions can minimize the experimental effort needed to identify new CTL epitopes to be used in for example vaccine design or for diagnostic purposes. Tong et al. [22] comments on the reports of algorithms that integrate MHC class I predictions with TAP and proteasomal cleavage specificities: "These techniques are still in their infancy and need to be further developed and thoroughly tested". Here, we make a first attempt to test the performance of five of these methods on two evaluation sets of experimentally verified HIV CTL epitopes. It turned out to be a highly non-trivial task to design an objective benchmark. Mainly because the prediction methods each generate epitope predictions in a specific format and potentially with different mechanisms that filter the number of prediction scores made available to the user. Our final performance measures consist firstly of a RANK measure that allows for an objective comparison of accuracy between the different prediction methods. For comparing prediction specificity, we define three levels of prediction sensitivity, so that comparisons can be performed at equal levels. Finally, we compare the sensitivity among the 5% top-scoring peptides as obtained by each method.

Using the defined performance measures, we performed a large-scale benchmark calculation comparing the predictive performance of a series of publicly available methods for CTL epitope prediction. The benchmark included the EpiJen, MAPPP, WAPP, and MHC-pathway methods, and an updated version of the NetCTL method. The updated version of NetCTL, version 1.2, can make predictions for the A26 and B39 HLA supertypes thus completing the list of 12 recognized supertypes, and was shown to have a higher predictive performance than the old version 1.0. We find that NetCTL-1.2 has a higher predictive performance than EpiJen, MAPPP, MHC-pathway, and WAPP on all measures. When comparing NetCTL-1.2 with MAPPP and WAPP, the higher performance of NetCTL-1.2 is statistically significant on all measures. When comparing NetCTL-1.2 with EpiJen, the higher performance of NetCTL-1.2 is statistically significant for all measures except when comparing the specificities at the sensitivity values of 0.3 and 0.5 on the HIV^EpiJen ^dataset. When comparing NetCTL-1.2 with MHC-pathway, the higher performance of NetCTL-1.2 is statistically significant for all measures, except when comparing the specificities at the sensitivity values of 0.3 and 0.5 on either evaluation dataset. It is not surprising that MHC-pathway reaches almost as high predictive performance as NetCTL-1.2 on some of the performance measures. These two methods have several features in common: Firstly, the MHC binding prediction methods included in the MHC-pathway and NetCTL prediction methods, have recently in a large scale benchmark been shown to have comparable performance [18]. Secondly, they use identical methods for predicting TAP transport efficiency; namely the matrix method developed by Peters et al. [23]. Thirdly, they integrate the predicted values obtained from the separate proteasomal cleavage, TAP transport efficiency, and MHC class I affinity predictors into one combined score. Regarding differences it can be mentioned that the proteasomal cleavage predictor used for MHC-pathway is trained on *in vitro *data, while NetCTL-1.2's proteasomal cleavage predictor, NetChop-3.0, is trained on natural MHC class I ligands.

NetCTL-1.2, MAPPP, and MHC-pathway integrates the predicted values into one, overall score, while EpiJen and WAPP use a number of successive filters that step by step reduce the number of possible epitopes. Doytchinova et al. [16] has stated that the "combined score as used by SMM (MHC-pathway) and NetCTL, obscures the final result, because a low (or even negative) TAP and/or proteasomal score could be compensated for by a high MHC score." We would here like to offer our interpretation of how the combined score can be understood in a biological meaningful manner: First of all, we see the predictive values as probabilities. Secondly, one has to keep in mind that there is not just one copy of a given protein in the cell. This means that if for example a certain peptide has a low predicted cleavage score and will only be generated in 1 out of a 100 cleavage events, the peptide can still survive all the way to the cell surface and become a CTL epitope, if the TAP transport efficiency and MHC class I affinity are sufficiently high.

We have throughout the analysis on the HIV dataset compared NetCTL-1.2 to each of the other test methods separately. This was done in order to include epitopes restricted to as many supertypes as possible. Had we chosen only to include epitopes restricted to supertypes that all methods had in common, we could only have included the A1, A2, and A3 supertypes. The shortcoming of this approach is that comparisons can not be made directly in between the test methods. For comparisons in between the test methods, we refer to calculations done on the HIV^EpiJen ^dataset, which only contains epitopes restricted to the A1, A2, and A3 supertypes.

Lastly, we would like to note that the NetCTL method predicts CTL epitopes that are presented via a pathway that utilizes TAP for peptide entry into ER. Additional pathways also exist as reviewed in [24]. Their contribution to the total presentation of MHC class I ligands is, however, thought to be minor [25-27].

## Conclusion

Using objective benchmarks and standardized performance measures, we have demonstrated that NetCTL-1.2 has a higher predictive performance than EpiJen, MAPPP, MHC-pathway, and WAPP, although when comparing NetCTL-1.2 with EpiJen and MHC-pathway, the higher predictive performance of NetCTL-1.2 is not statistically significant on some of the measures.

The benchmark datasets are all available and downloadable from the Internet. Together with the detailed description on how to perform the calculations and extract the different performance measures presented here, it is our hope that other researches readily can repeat the benchmark analysis, and in an objective manner compare novel methods for CTL epitope discovery to the five methods included here.

## Methods

### Datasets

#### Training set

In August 2006, 1730 9 meric peptides present in the SYFPEITHI database [12,28] and listed as either "Example for Ligand" or "T-cell epitope" were extracted. The peptides were grouped according to MHC class I allele and one of the 12 supertypes as defined in [20]. Peptides that had been used to develop one or more of the methods for predicting proteasomal cleavage, TAP transport efficiency, or MHC class I affinity for NetCTL-1.2 were removed. Then, for every peptide, the source protein was found in the SwissProt database. If more than one source protein was possible, the longest human protein was chosen, and if there were no possible human proteins, the longest other protein was chosen. The final SYFPETHI dataset contained a total of 863 epitope-protein pairs. All 9 meric peptides contained in the source protein sequences, except those annotated as epitopes in either the complete SYFPEITHI or Los Alamos HIV databases [29], were taken as negative peptides (non-epitopes). When using this definition of epitope/non-epitope one has to take into account that some epitopes will falsely be classified as non-epitopes because the SYFPEITHI and Los Alamos HIV databases are incomplete. Since the MHC class I molecules are very specific, binding only a highly limited repertoire of peptides, this misclassified proportion will, however, be very small. A given MHC class I molecule has a specificity of ~1% [9]. In a protein of 100 amino acids, one expects to have one binding and 99 non-binding peptides. The potential number of false classifications is hence orders of magnitudes smaller than the actual number of negatives. Furthermore, the measured performance of all the methods should be equally affected by the false negatives in the dataset, thus while the reported absolute performance of the methods might be underestimated, we do not expect the false negatives to affect the relative ranking of the different methods.

This dataset will hereafter be referred to as the SYFPEITHI dataset.

#### Evaluation sets

In December 2005, 342 9 meric peptides present in the HIV Immunology CTL database of the Los Alamos HIV Database [29] were extracted. The peptides were subsequently sorted as for the SYFPEITHI dataset. In all, the epitopes in the final dataset are restricted to 29 MHC class I alleles that can be further divided into one of the 12 recognized supertypes [20]. Subsequently, the source proteins were found. If more than one protein was the possible origin of a given peptide, the longest protein was chosen. The final Los Alamos HIV dataset contained 216 epitope-protein pairs. All 9 meric peptides contained in the source protein sequences, except those annotated as epitopes in either the complete SYFPEITHI or Los Alamos HIV databases, were taken as negative peptides (non-epitopes). The dataset will hereafter be referred to as the HIV dataset. Another evaluation set was taken from [16]. This dataset was compiled from the Los Alamos HIV database in June 2005, but contained only epitopes restricted to the A1, A2, or A3 supertypes. Originally it contained 99 epitopes, but we removed 12 of them, since they had been used previously to train NetCTL-1.2. For the 87 remaining peptides, the source proteins were subsequently found. If more than one protein was the possible origin of a given peptide, the longest protein was chosen. The final dataset, which is called HIV^EpiJen^, thus contains 87 epitope-protein pairs. It may be noted, that this approach differs from the one used in [16], where all epitopes are mapped to the HXB2 consensus protein sequence. All 9 meric peptides contained in the source protein sequences, except those annotated as epitopes in either the complete SYFPEITHI or Los Alamos HIV databases, were taken as negative peptides (non-epitopes). In summary, the HIV and HIV^EpiJen ^datasets are both compiled from the Los Alamos HIV database, but whereas the HIV dataset contains 216 epitopes restricted to all 12 recognized supertypes, the HIV^EpiJen ^dataset contains 87 epitopes restricted to only the A1, A2, or A3 supertype. The HIV dataset was compiled by ourselves, while the HIV^EpiJen ^dataset was taken from [16]. Of the 87 epitopes in the HIV^EpiJen ^dataset, 59 are also present in the HIV dataset [21].

All above mentioned datasets are available as supplementary material at [21].

### Prediction methods

#### NetCTL-1.2

Prediction of proteasomal cleavage patterns was done by the NetChop 3.0 method [30,31], which is an artificial neural network (ANN) trained on natural MHC class I ligand data. Prediction of TAP transport efficiency was performed using the matrix method described by Peters et al. [23]. For MHC class I affinity predictions, we use an updated version of the method described by Nielsen et al. [32] (NetMHC-3.0^NO_HIV^) and include all 12 supertypes: A1, A2, A3, A24, A26, B7, B8, B27, B39, B44, B58, and B62 [20]. For training of NetMHC-3.0^NO_HIV ^HIV data were excluded, but otherwise the method is identical to the method available at [33]. This was done in order to retain the maximal size of the evaluation sets, which only consist of HIV data. Note that the NetCTL-1.2 method available at [34] integrates the complete NetMHC-3.0 method. Table 3 shows which alleles are used to represent each of the 12 supertypes. The weights on proteasomal cleavage and TAP predictions are determined in a five-fold cross validated manner optimizing the predictive performance on the SYFPEITHI dataset. For each of the epitope/protein pairs in the training set, the AUC is calculated and the set of weights on proteasomal cleavage and TAP prediction that achieves optimal mean AUC value are identified. Optimal weights on cleavage and TAP transport were found to be 0.15+/-0.01, and 0.05 +/- 0.01, respectively, with an average AUC performance of 0.975 over the 863 epitope/protein pairs. The output from NetCTL-1.2 is a single, combined score for every possible peptide in a given protein.

**Table 3:** Representative alleles

Supertype	NetCTL	EpiJen	MAPPP	MHC-pathway	WAPP
A1	HLA-A*0101	HLA-A*0101	HLA-A1	HLA-A*0101	HLA-A*01
A2	HLA-A*0201	HLA-A*0201	HLA-A*0201	HLA-A*0201	HLA-A*0201
A3	HLA-A*0301	HLA-A*0301	HLA-A3	HLA-A*0301	HLA-A*03
A24	HLA-A*2402	HLA-A*24	HLA-A24	HLA-A*2402	N/A
A26	HLA-A*2601	N/A	N/A*	HLA-A*2601	N/A
B7	HLA-B*0702	HLA-B*07	HLA-B7	HLA-B*0702	N/A
B8	HLA-B*0801	N/A	HLA-B8	HLA-B*0801	N/A
B27	HLA-B*2705	HLA-B*27	HLA-B*2705	HLA-B*2705	HLA-B*2705
B39	HLA-B*3901	N/A	HLA-B*3901	N/A	N/A
B44	HLA-B*4001	HLA-B*40	HLA-B40	HLA-B*4002	N/A
B58	HLA-B*5801	N/A	HLA-B*5801	HLA-B*5801	N/A
B62	HLA-B*1501	N/A	HLA-B62	HLA-B*1501	N/A
# epitope-protein pairs	216	188	214	215	131

The table shows which alleles are used for representing the supertypes in the HIV and HIV^EpiJen ^datasets. The first column gives the HLA supertype, the next five columns give the alleles used a supertype representatives for each of the five prediction method NetCTL-1.2, EpiJen, MAPPP, MHC-pathway, and WAPP, respectively. The lower row (N) gives the total number of epitope-protein pairs in the HIV dataset covered by each of the five prediction methods. *A MHC type termed HLA-A26 was listed, but did not produce any results.

#### EpiJen [16]

Like NetCTL-1.2, MHC-pathway, and WAPP, this algorithm operates with three steps in order to predict CTL epitopes: Proteasomal cleavage, TAP transport, and MHC class I binding. Each step is based on a quantitative matrix and acts as a filter that reduces the number of potential epitopes. The method is available at [35] and includes CTL epitope predictions for 18 different alleles. Table 3 shows which alleles we use to represent the supertypes in the HIV and HIV^EpiJen ^dataset. No alleles can represent the A26, B39, B58, or B62 supertypes. When calculating the performance measures for EpiJen on the HIV dataset, we therefore only have a total of 188 epitope-protein pairs as compared to 216 epitope-protein pairs for all 12 supertypes. Different cut offs can be chosen for the proteasomal cleavage and TAP transport filters. In each case, we used the recommended cut offs. The final scores are the predicted MHC class I affinities in form of -logIC_50 _and IC_50 _values. It is not possible to retrieve scores for all possible peptides in a given protein – at most, the EpiJen server outputs the 5% peptides that have the highest predicted MHC class I affinity and at the same time pass the proteasomal cleavage and TAP transport filters.

#### MAPPP [15,36]

Unlike the other four methods, MAPPP only operates with proteasomal cleavage and MHC class I binding. Proteasomal cleavage can be done by either the FRAGPREDICT [37,38] or PAProC [39,40] method. For this study we chose FRAGPREDICT, since it is the default choice. MHC class I binding can be done by either the SYFPEITHI Epitope Prediction method [11] or the BIMAS HLA Peptide Binding Prediction method [13]. We used the BIMAS HLA Peptide Binding Prediction method, since we have previously found this method to be superior [10]. Binding to the A26 supertype was listed to be done only by the SYFPEITHI Epitope Prediction method, but in spite or several attempts, we never received any results for this supertype. Consequently, we left out this supertype when doing calculation for the MAPPP method on the HIV dataset. Table 3 shows which alleles we use to represent the remaining supertypes in the HIV and HIV^EpiJen ^dataset. Excluding the A26 supertype, we have a total of 214 epitope-protein pairs for 11 supertypes in the HIV dataset. The output is a combined score from the proteasomal cleavage and MHC class I binding predictions. It is possible to retrieve scores for all peptides in a given protein.

#### MHC-pathway [17,18]

As NetCTL-1.2, MHC-pathway integrates the scores obtained from three methods predicting, respectively, proteasomal cleavage, TAP transport, and MHC class I affinity into one final score. The method for predicting proteasomal cleavage is a matrix-based algorithm called the Stabilized Matrix Method (SMM) trained on *in vitro *cleavage data. The method for predicting TAP transport efficiency is identical to the one used for NetCTL-1.2 and is described in [23]. The method for predicting MHC class I affinity is also based on a SMM. The original MHC-pathway method [17] is available from [41], while an updated version of the method is located at [42]. In this study we have used the updated version. It is possible to predict CTL epitopes restricted to 34 different human alleles. Table 3 shows which alleles we use to represent the supertypes in the HIV and HIV^EpiJen ^dataset. No alleles can represent the B39 supertype. When calculating the performance measures for MHC-pathway on the HIV dataset, we therefore only have a total of 215 epitope-protein pairs as compared to 216 epitope-protein pairs for all 12 supertypes. We used default settings for proteasomal cleavage (immuno proteasome) and TAP transport predictions. In the final output, MHC-pathway provides a single, combined score for all possible peptides in a given protein.

#### WAPP [19]

Like NetCTL-1.2, EpiJen, and MHC-pathway, this algorithm operates with predictions for proteasomal cleavage, TAP transport, and MHC class I affinity. The proteasomal cleavage predictor employs a matrix-based method trained on experimentally verified proteasomal cleavage sites. Support vector regression is used for predicting peptides transported by TAP. MHC class I affinity is predicted using a support vector machine. Each step acts as a filter that reduces the number of potential epitopes. The method is available at [43] and includes predictions for HLA-A*01, HLA-A*0201, HLA-A*03, and HLA-B*2705. Table 3 shows which alleles we use to represent the supertypes in the HIV and HIV^EpiJen ^dataset. No alleles can represent the A24, A26, B7, B8, B39, B44, B58, or B62 supertypes. When calculating the performance measures for WAPP on the HIV dataset, we therefore only have a total of 131 epitope-protein pairs as compared to 216 epitope-protein pairs for all 12 supertypes. It is possible to retrieve predicted values for proteasomal cleavage, TAP transport, and MHC class I affinity for all possible peptides in a protein. The proteasomal cleavage and TAP transport filters can be set at different levels between 1 and 5. We used the default levels, which for both filters are 3. These levels correspond to a predicted proteasomal cleavage value above -1.2 and a predicted TAP transport value below -37.5 (as kindly informed by Pierre Dönnes). Prediction scores for all methods and for all nonamers are available as supplementary material [21].

### Performance measures

#### Sensitivity and specificity

The formulas for calculating sensitivity and specificity are listed below:

Sensitivity = TP/AP

Specificity = TN/AN

Where

TP = true positives, which are the correctly predicted epitopes in the dataset, AP = actual positives, which are the actual number of epitopes in the dataset, TN = true negatives, which are the correctly predicted non-epitopes in the dataset, AN = actual negatives, which are the actual number of non-epitopes in the dataset.

#### AUC

The AUC value (the Area Under the ROC Curve) is calculated per epitope-protein pair. All overlapping 9 meric peptides in the protein are sorted according to the predicted score. For NetCTL-1.0, NetCTL-1.2, MAPPP, and MHC-pathway, the predicted score is combined from the predicted proteasomal cleavage, TAP transport, and MHC class I affinity values. For WAPP it is the predicted MHC class I affinity for peptides that pass the proteasomal cleavage and TAP transport filters. For EpiJen, the predicted score is also the predicted MHC class I affinity, but is only available for the 5% peptides that have the highest predicted MHC class I affinity, and which at the same time pass the proteasomal cleavage and TAP transport filters. The epitopes in the epitope-protein pairs define the positive set, whereas the negative set is made up from all other 9 mers in the source proteins excluding 9 mers found in the complete SYFPHITHI or Los Alamos HIV databases. The ROC curve is plotted from the sensitivity and 1-specificity values calculated by varying the cut-off value (separating the predicted positive from the predicted negative) from high to low. The area under this curve gives the AUC value. The AUC value is 0.5 for a random prediction method and 1.0 for a perfect method. When comparing the predictive performance (measured by AUC) of two prediction methods, a paired t-test is applied to test whether the observed difference in average AUC values differs significantly from zero.

#### RANK

Two methods at a time are compared by this performance measure. The two methods are NetCTL-1.2 and one of the four test methods (EpiJen, MAPPP, MHC-pathway, or WAPP). Calculations are done on the HIV and HIV^EpiJen ^datasets separately. For comparison on the HIV dataset, we only include epitope-protein pairs, where the epitope is restricted to a supertype covered by the test method. To facilitate comparison to the EpiJen and WAPP methods, where only a subset of the peptides are assigned a predicted value, only the top N of the NetCTL-1.2 predictions where included, where N is the number of peptides predicted by the test method (EpiJen or WAPP). All peptides without a predicted value are assigned the rank 9999 to put them at the bottom of the rank-list. In this way, all methods are compared on an equal number of peptide data. For MAPPP and MHC-pathway all peptides are included. We next count how often NetCTL-1.2 ranks the epitope higher than the test method, and vice versa. For all comparisons, all epitopes in either the complete SYFPEITHI or Los Alamos HIV databases are disregarded, except for the particular epitope belonging to the epitope-protein pair in question. When comparing the predictive performance (as measured by RANK) of NetCTL-1.2 and the test method, we examine whether the observed higher proportion of proteins for which NetCTL-1.2 ranks the epitope highest deviates significantly from what is expected under a binomial distribution, where both methods have a probability of 0.5 for ranking the epitope highest. Proteins for which the methods rank the epitope equally high are omitted from the analysis.

#### Specificity at a predefined sensitivity

When using the HIV dataset for the analysis, two methods at a time are compared by this measure: NetCTL-1.2 and one of the four test methods (EpiJen, MAPPP, MHC-pathway, or WAPP). We only include epitope-protein pairs, where the epitope is restricted to supertypes covered by the test method. All calculations are made per epitope-protein pair, which means that for a given epitope-protein pair the sensitivity will either be 1 (the epitope is identified at the given threshold) or 0 (the epitope is not identified at the given threshold). First, for every method three threshold values in the form of combined scores (NetCTL-1.2, MAPPP, and MHC-pathway) or predicted MHC class I affinities (EpiJen and WAPP) are identified, which achieve a sensitivity of 0.3, 0.5, or 0.8, when averaging over all epitope-proteins pairs. Notice that EpiJen and WAPP do not provide enough predicted scores to achieve a sensitivity of 0.8. Due to the different size of the HIV dataset depending on the test method in question, three different thresholds values are found for NetCTL-1.2 when compared to either of the test methods. Next, the specificity is calculated per epitope-protein pair using the same threshold values. For the HIV^EpiJen ^datasets all methods cover all epitopes. Again, three threshold values are found for each method and the specificity is calculated per epitope-protein pair using the same threshold values. An unpaired student's t-test [44] is applied to test whether the average specificity of NetCTL-1.2 at a given sensitivity is significantly different from the average specificity at the same sensitivity for a test method.

#### Sensitivity among the 5% top-scoring peptides

When using the HIV dataset, two methods at a time are compared by this measure: NetCTL-1.2 and one of the four test methods. We only include epitope-protein pairs, where the epitope is restricted to supertypes covered by the test method. For the HIV^EpiJen ^datasets all methods cover all epitopes. For calculating the sensitivity among the top 5% peptides, we rank all possible 9 mers for the proteins in the dataset in question according to the combined score (NetCTL-1.2, MAPPP, and MHC-pathway) or according to the predicted MHC class I affinity (EpiJen and WAPP). We only operate with one epitope per protein and accordingly remove all other known epitopes from the SYFPEITHI or Los Alamos HIV databases from the protein in question (all known epitopes from the SYFPEITHI or Los Alamos HIV databases are listed per supertype as supplementary material [21]). Finally, we calculate the sensitivity among the 5% top-scoring peptides.

## Authors' contributions

MVL contributed to the design of the study, compiled the datasets, obtained the data for the MAPPP, MHC-pathway, and WAPP methods, analysed the data for the EpiJen, MAPPP, MHC-pathway, and WAPP methods, participated in the design of the NetCTL method, and drafted the manuscript. CL contributed to the design of the study, participated in the design of the NetCTL method, and obtained the data for the EpiJen method. KL generated data used for the NetCTL method. SB contributed to the design of the study and generated data used for the NetCTL method. OL contributed to the design of the study and participated in the design of NetCTL. MN contributed to the design of the study, participated in the design of the NetCTL method, implemented the NetCTL method, analysed the data for the EpiJen, MAPPP, MHC-pathway, and WAPP methods, and helped drafting the manuscript. All authors read and approved the manuscript.
